# Negative Diffusion Weighted Imaging on Magnetic Resonance Imaging of the Brain in Creutzfeldt–Jakob Disease

**DOI:** 10.1155/2020/8857037

**Published:** 2020-12-21

**Authors:** Elijah Lackey, Deepal P. Shah-Zamora, Jodi Hawes, Andy J. Liu

**Affiliations:** Duke University Department of Neurology, Duke University Medical Center 2905, 40 Medicine Circle, Durham, NC 27710, USA

## Abstract

A 76-year-old Caucasian woman initially presented to the Duke Memory Disorders clinic with a 9-month history of a rapid decline in cognitive, motor, and neuropsychiatric function. On initial presentation, the patient required assistance with activities of daily living. On neurological examination, she was found to have Gerstmann's syndrome along with appendicular apraxia. A positional tremor was noted without myoclonus or fasciculations. She had a paucity of speech and was unable to write her own name. Snout and grasp reflexes were present. Episodes of inappropriate laughter were noted during the exam. She was admitted to the inpatient neurology service for further evaluation. The Diffusion Weighted Imaging sequence on Magnetic Resonance Imaging of the brain was negative for restricted diffusion. An electroencephalogram was unremarkable. Cerebrospinal fluid analysis for Real-Time Quaking-Induced Conversion assay was positive along with an elevated 14-3-3 and increased total Tau protein levels. There was no family history of Creutzfeldt–Jakob disease. The cerebral spinal fluid results were consistent with a diagnosis of Creutzfeldt–Jakob disease, despite the negative MRI brain findings.

## 1. Introduction

Creutzfeldt–Jakob Disease (CJD) is commonly described as a rapidly progressive dementia with a subacute onset. The disease presents with a heterogeneous phenotype and may include progressive cognitive, behavioral, motor, and sensory deficits. Higher-order cortical deficits may be present in about 10–15% of cases as early symptoms and in about half of patients as the disease progresses [1]. A common biomarker of CJD is restricted diffusion on the Diffusion Weighted Imaging (DWI) sequence on Magnetic Resonance Imaging (MRI) brain localized to the neocortex, basal ganglia, and caudate [1–3]. A Fluid-Attenuated Inversion Recovery (FLAIR) or T2 sequence hyperintensity correlating with the neocortical restricted diffusion on DWI may also be seen [1, 3]. An electroencephalogram (EEG) may show 1-2 Hz periodic sharp wave complexes [1, 3]. Restricted diffusion on the DWI sequence has a sensitivity of 96% in patients with CJD [1–3]. This case report underscores the importance of pursuing a comprehensive evaluation including cerebrospinal fluid (CSF) analysis, MRI of the brain, and an EEG in all patients with subacute changes in behavior, motor function, or cognition.

## 2. Case Presentation

A 76-year-old female with a history of hypertension, hyperlipidemia, and coronary artery disease was referred to the Duke Memory Disorders clinic by her local neurologist for evaluation of a rapid decline in cognitive, behavioral, and motor symptoms over a 9-month period. She had no family history of neurodegenerative disease. Nine months prior to presentation, she experienced two myocardial infarctions that required two cardiac catheterization procedures. Before these procedures, family members stated that she lived independently. After her first cardiac catheterization, she began having progressive language difficulties. Evaluation by speech therapy was notable for difficulty in completing written assignments and a decrease in spontaneous speech. The patient was also noted to have executive dysfunction such as having challenges managing the family's finances or placing an empty pot on the stove with the burner on, which she could previously do independently. Behavioral changes were also appreciated by the family such as easily becoming upset and crying frequently, and she had become more agreeable after her cardiac procedures. She also became less energetic and spent more time sleeping. She then developed motor difficulties described as unsteadiness while walking, particularly going up stairs, and later had difficulty navigating her home.

On general examination in clinic, her vital signs were unremarkable. Mental status examination was notable for a paucity of speech and inability to write her own name. There were episodes of inappropriate laughter throughout the exam. Neurological examination revealed appendicular apraxia, an action tremor, along with snout and grasp reflexes. No myoclonus or fasciculations were noted. All elements of Gerstmann's syndrome were appreciated including finger agnosia, right-left confusion, agraphia, acalculia, and abulia.

**Figure 1:**
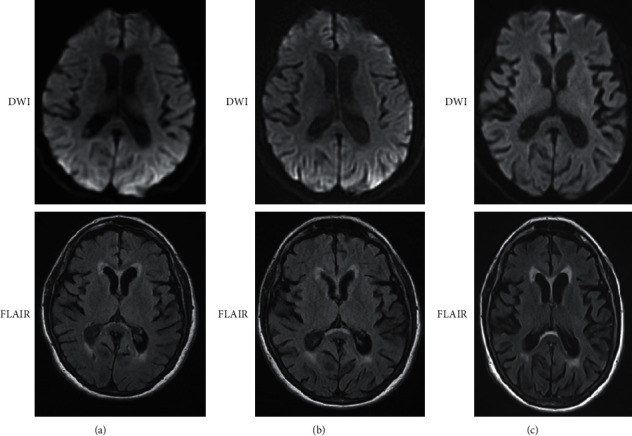
Axial MRI diffusion weighted images (top row) and fluid-attenuated inversion recovery images shown at about one month (a), four months (b), and nine months (c) from onset of symptoms. These images show no diffusion restriction on diffusion weighted images or hyperintensities on FLAIR that would support the diagnosis of Creutzfeldt–Jakob disease.

**Figure 2:**
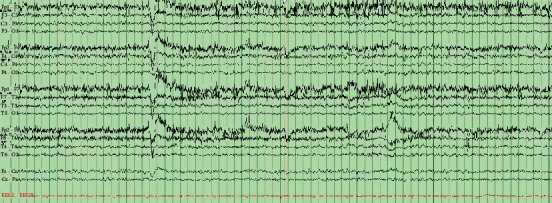
Electroencephalogram (EEG) completed at about nine months from the onset of symptoms. The occipital dominant rhythm was 9 Hz. EEG activity was reactive to stimulation. There were no 1-2 Hz periodic sharp wave complexes suggestive of Creutzfeldt–Jakob disease.

MRI brain at one month after symptom onset showed no diffusion restriction (Figure 1). A Montreal Cognitive Assessment (MoCA) completed 3 months after her cardiac procedures was 18/30. The test was repeated 1 month later with a total score of 14/30. Three months after onset of symptoms, a routine EEG was ordered by the local neurologist which was unrevealing (Figure 2). Labwork including basic metabolic panel, complete blood count, urinalysis, and urine culture were normal. Four months after onset of symptoms, a repeat MRI showed no significant changes from the prior brain MRI.

Given concerns for CJD after the initial evaluation at our institution's memory disorders clinic, the patient was directly admitted to the inpatient neurology service for expedited evaluation. A repeat routine EEG and MRI brain were both unrevealing. CSF RT-QuiC assay and 14-3-3 protein was positive. The t-tau level was 1891 pg/ml. Collectively, the CSF results were consistent with a diagnosis of CJD (Table 1).

## 3. Discussion

CJD is difficult to diagnose because of its heterogeneous clinical phenotype. The heterogeneity is dependent on the anatomic area of involvement of the prion protein along with the histopathologic subtype. Histopathologic subtypes of spontaneous CJD are based on the polymorphism in the prion gene, PRNP, at codon 129 (methionine or valine) paired with the molecular weight of the PrP^SC^ protein as run on western blot [4, 5]. Subsequently, there are six possible combinations of polymorphisms paired with molecular weight (MM1, MV1, VV1, VV2, MM2, and MV2), which can be further subdivided based on location (MM2 cortical vs. MM2 thalamic), although sometimes both molecular weights may be present (MM1-2, MV1-2, and VV1-2). Comprehensive characterization of these subtypes has revealed stereotypical clinical phenotypes [6]. Nevertheless, histopathologic subtypes do not necessarily correlate with clinical phenotype, and diagnosis may not be straightforward [4]. Subsequently, a comprehensive evaluation including MRI of the brain, EEG, and CSF analysis are required for the diagnosis of CJD [7, 8]. Overall, MRI brain has 92–96% sensitivity for diagnosis of CJD although accuracy of the test depends on histopathologic subtype with striatal or cortical ribbon diffusion seen more often in common subtypes such as MM1 and MV1 [3, 4, 7, 9]. In contrast, the rare MM2 cortical and VV1 subtypes often do not show any MRI findings, and it is possible that our patient may have had one of these variants [10, 11]. The EEG has 60–80% sensitivity with higher sensitivity later in the disease course [12–14]. The RT-QuiC test combined with 14-3-3 has an estimated sensitivity of 77–92% and specificity of 99–100% making it highly useful as a diagnostic test if positive [15, 16]. Serologic codon 129 polymorphisms can now be identified with antemortem genetic testing which is useful for diagnostic confirmation and for assessing for inherited disease.

This case report highlights the importance of the clinical trajectory in patients with a rapidly progressive dementia. Three DWI sequences from the MRI brain studies were negative for restricted diffusion. Intriguingly, the CSF biomarkers supported the diagnosis of CJD. Based on the CSF RT-QuiC, t-tau, and 14-3-3 protein assays, the National Prion Disease Pathology Surveillance Center reported a likelihood of CJD of greater than 98%. Currently, the treatment for CJD is supportive.

Definitive diagnosis of CJD by WHO guidelines is made by postmortem autopsy. Genetic testing is performed on brain tissue obtained at biopsy. At this time, the patient is enrolled in Home Hospice; therefore, her histopathologic subtype and genotype of PRNP is unknown. Antemortem genetic testing was discussed with the daughter but deferred. In conclusion, if initial testing is discordant with the clinical presentation as was in this case, a comprehensive evaluation for a rapidly progressive dementia should be pursued.

## Figures and Tables

**Table 1:** Cerebrospinal fluid (CSF) studies sent approximately nine months from onset of symptoms along with serum studies completed throughout the clinical course.

Test (source)	Result	Normal reference range
RT-QuIC (CSF)	Positive	Negative
14-3-3 protein (CSF)	Positive	Negative
Total tau protein (CSF)	1891 pg/ml	0–1149 pg/ml
Nucleated cells (tube #4 CSF)	1/*μ*L	0–5/*μ*L
Red blood cells (tube #4 CSF)	13	<=0/*μ*L
Glucose (CSF)	64 mg/dl	60% of plasma level (90 mg/dl)
Protein (CSF)	39 mg/dl	15–50 mg/dl
CSF and serum autoimmune panels	Negative for autoantibodies	Negative for autoantibodies
VDRL (CSF)	Negative	Negative
TSH (serum)	1.49 *μ*LU/ml	0.358–3.74 *μ*LU/ml
Vitamin B12 (serum)	625 pg/ml	211–911 pg/ml
Antithyroglobulin (TPG) antibody (serum)	<1	< or = 1 IU/mL
Antithyroperoxidase (TPO) antibody (serum)	<1	<9 IU/mL
Paraneoplastic panel (serum)	Negative for autoantibodies	Negative for autoantibodies

## Data Availability

Data used to support the findings of this study can be obtained from the corresponding author upon request.
